# *Bjerkandera adusta* Fungi as Causative Agent of Invasive Chronic Rhinosinusitis

**DOI:** 10.3201/eid3102.241275

**Published:** 2025-02

**Authors:** Yuhei Kurata, Yoshifumi Kimizuka, Takashi Yaguchi, Kanshu Ito, Tetsuya Yamamoto, Yusuke Serizawa, Akira Kamiya, Takaaki Hamamoto, Taishi Sakima, Tomomi Tanigaki, Hiromi Edo, Yu Hongo, Akira Watanabe, Kazushi Suzuki, Terushige Toyooka, Akihiko Kawana

**Affiliations:** Author affiliations: National Defense Medical College, Saitama, Japan (Y. Kurata, Y. Kimizuka, K. Ito, T. Yamamoto, Y. Serizawa, T. Sakima, T. Tanigaki, H. Edo, Y. Hongo, K. Suzuki, T. Toyooka, A. Kawana); Chiba University, Chiba, Japan (T. Yaguchi, A. Watanabe); National Defense Medical College Hospital, Saitama (A. Kamiya, T. Hamamoto)

**Keywords:** Bjerkandera adusta, fungi, sinusitis, voriconazole, biopsy, diabetes mellitus, case report, fungal infection, rhinosinusitis, mycosis

## Abstract

We report an invasive mycosis case in Japan caused by *Bjerkandera adusta*, a fungal species not previously reported as a causative pathogen of invasive mycosis. *B. adusta* was identified by using phylogenetic analysis. Voriconazole was used successfully for treatment. Immunodeficient patients may be susceptible to infection by rare causative fungi.

Sinonasal mycosis is a concerning deep-seated fungal disease. Invasive sinonasal mycosis is known for its poor prognosis and propensity for intracranial and intraorbital complications that are characterized by bone-destructive progression in immunodeficient conditions, including poorly managed diabetes, malignancies, long-term use of glucocorticoids and immunosuppressive drugs, and AIDS (*1*,*2*). The most common causative fungi are *Aspergillus* and *Mucor* spp.; however, other fungi, such as *Alternaria*, *Scedosporium*, *Candida*, and *Fusarium* spp. have been reported (*3*). In this article, we report the clinical course of an invasive mycosis case in Japan in which *Bjerkandera adusta*, a white-rot fungus that typically occurs on dead trees and stumps in forests, was identified in a patient biopsy specimen of a lesion. The patient provided written consent for the publication of this case report.

## The Case

**Figure 1:**
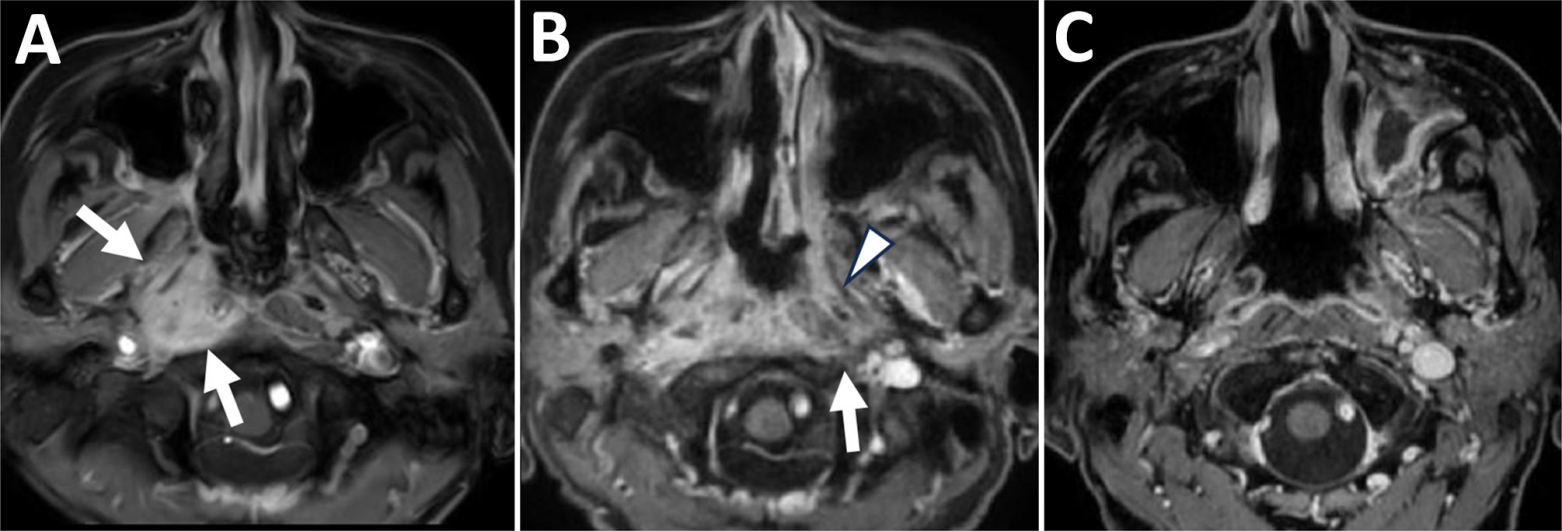
Chronological changes in lesions observed on contrast-enhanced fat-suppressed T1-weighted magnetic resonance imaging at the nasopharyngeal level in a patient in Japan with invasive chronic rhinosinusitis caused by *Bjerkandera adusta* fungi. A) Initial visit. Enhancement effects observed in the right nasopharynx, including the right torus tubarius and right prevertebral space (white arrows). B) Three months after the initial visit. Expansion of the enhancing lesion is seen, with enhancement extending to the left peritubal region (white arrowhead) and prevertebral space (white arrow). C) One year after the initial visit. The abnormal enhancement previously observed around both the torus tubarius and prevertebral space has regressed.

In November 2021, a 57-year-old woman with a history of type 2 diabetes and diabetic retinopathy experienced a headache that radiated throughout her head (numeric rating scale [NRS] 10) and a painful sensation in the right side of her face, radiating from under the jaw to the cheek. She was a housewife without any specific hobbies outside the home. We conducted magnetic resonance imaging (MRI) in February 2022 to investigate the cause of the symptoms. The MRI revealed a mild subacute cerebral infarction in the right parietal lobe but no other obvious abnormalities. The symptoms persisted despite cilostazol treatment. We conducted a repeat MRI in August 2022 that revealed a mass-like lesion near the right cavernous sinus. Extensive infiltrative growth patterns were found in the right sphenoid sinus, right orbital apex, right cavernous sinus, dura mater inside the right middle cranial fossa, right side of the clivus, right skull base, right temporalis muscle, right levator palatopharyngeus muscle, and soft tissue around the right eustachian tube (Figure 1, panel A).

**Figure 2:**
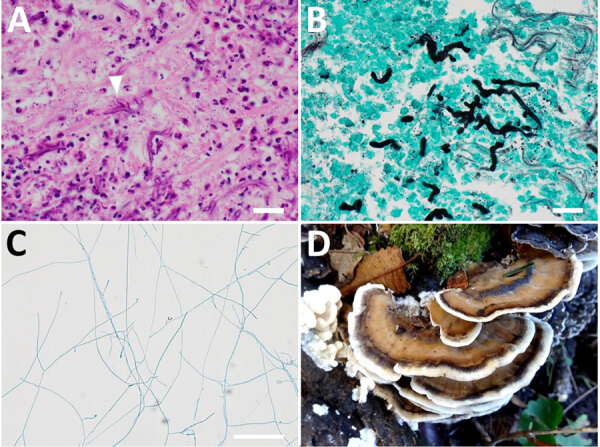
Imaging from a biopsy specimen from a sinus lesion in a patient in Japan with invasive chronic rhinosinusitis caused by *Bjerkandera adusta* fungi and a *Bjerkandera* spp. mushroom found in nature. A) Mycelium of filamentous fungi (arrowhead) shown by hematoxylin-eosin staining. Scale bar indicates 20 μm. B) Filamentus fungi shown by Grocott staining. Scale bar indicates 20 μm. C) Mycelium of fungi grown on potato dextrose agar. Scale bar indicates 100 μm. D) *Bjerkandera* spp., a burnt-looking stemless mushroom that grows in forests. Photograph courtesy of the Tokyo Mushroom Club.

Laboratory testing revealed an elevated erythrocyte sedimentation rate and unremarkable β-D-glucan levels, except for a slight elevation near the upper reference limit (Table 1). Cerebrospinal fluid analysis revealed elevated protein levels but unremarkable culture and cell count (Table 2). We conducted a transnasal biopsy of deep tissue from the right skull base with aseptic exposure of the orbital floor on August 26, 2022. Hematoxylin and eosin staining and Grocott methenamine silver staining of the tissue revealed branched, funguslike structures (Figure 2, panels A, B). However, cultures on bromothymol blue agar, sheep blood agar, and chocolate agar were negative, and the fungal species could not be identified. On October 13, we conducted an additional biopsy and curettage of the lesion. Cultures on potato dextrose agar grew filamentous fungi (Figure 2, panel C), and the samples were sent to the Chiba University Mycology Center (Chiba, Japan) for species identification. The fungus was identified as *B. adusta* by using phylogenetic analysis (Appendix).

**Table 1:** Blood test results obtained from a patient with *Bjerkandera adusta*–caused invasive chronic rhinosinusitis, August 2022

Test	Value
Total bilirubin, mg/dL	0.53
Aspartate aminotransferase, U/L	10
Alanine aminotransferase, U/L	7
Lactate dehydrogenase, U/L	202
Blood urea nitrogen, mg/dL	19
Creatinine, mg/dL	0.97
Sodium, mmol/L	137
Potassium, mmol/L	4.5
Chloride, mmol/L	99
C-reactive protein, mg/dL	0.8
Erythrocyte sedimentation rate, mm/h	105
β-D glucan, pg/mL	19
*Candida* antigen, U/mL	<0.05
*Aspergillus* antigen cutoff index	0.3
*Cryptococcus* antigen	Negative
Leukocyte, 10^3^ cells/µL	9.4
Neutrophils, %	55.6
Lymphocytes, %	39
Monocytes, %	3.8
Eosinophils, %	1.4
Basophils, %	0.2
Hemoglobin, g/dL	10.2
Platelet, 10^3^/µL	480

**Table 2:** Cerebrospinal fluid test results obtained from a patient with *Bjerkandera adusta*–caused invasive chronic rhinosinusitis, August 2022

Test	Value
Cell count/μL*	4
Specific gravity	1.006
pH	8.2
Protein, mg/dL	147
Glucose, mg/dL	92
Sodium, mmol/L	148
Potassium, mmol/L	3
Chloride, mmol/L	127
Albumin, g/dL	708.9
IgG, mg/dL	19.5
IgA, mg/dL	6.46
IgM, mg/dL	0.58

*Cell fractionation not detected.

While waiting for the species to be identified, we initiated liposomal amphotericin B (L-AMB; 5 mg/kg/d) treatment on October 19, 2022, for invasive sinonasal fungal disease. However, on November 1, an MRI revealed that the contrast-enhanced lesion had spread to the left peritubal region and prevertebral space (Figure 1, panel B). Because of drug-induced renal damage, we added posaconazole (50 mg 3×/d) to the treatment regimen and performed a local nasal lavage on November 8. On the same day, the patient was reported to have a fever. We suspected a central venous catheter–related bloodstream infection and fever caused by posaconazole. Therefore, we discontinued posaconazole, initiated meropenem and vancomycin, and continued nasal lavage with the antifungal drug. On November 14, the fungal species was reported as *B. adusta*. On the basis of previously reported in vitro drug susceptibility data (*4*), we initiated voriconazole on December 7. The headache, initially rated at NRS 7–10, showed improvement beginning December 18, with a notable reduction to NRS 1. On December 26, an MRI revealed that the lesion expansion had stopped, with progression suppressed. We administered voriconazole intravenously for 3 weeks and then switched to oral administration on January 1, 2023, for a total treatment duration of 6 months. Blood β-D-glucan levels peaked at 52 pg/mL 4 months after the initial examination and decreased over time, returning to unremarkable levels by March 2023. We observed a gradual decrease in the abnormal contrast enhancement on MRI in March and July (Figure 1, panel C).

*B*. *adusta* is a white-rot fungus belonging to the phylum Basidiomycota, which forms mushrooms ≈4 inches in diameter (Figure 2, panel D). The mushrooms are widely distributed in temperate and subtropical zones worldwide and grow on dead or stunted broad-leaved trees. In humans, spores of this fungus act as allergens in the respiratory system, causing chronic cough and throat discomfort (*5*). Together with other basidiomycetes, this condition is known as fungus-associated chronic cough (*6*). We were unable to find other reported cases of invasive sinusitis caused by *B*. *adusta*. The patient lives in a commuter town adjacent to Tokyo, which is relatively warm and humid throughout the year and is surrounded by woods, a habitat for *B. adusta* that might have led to this infection.

## Conclusions

In invasive fungal sinusitis, such as mucormycosis and aspergillosis, the fungus invades the blood vessels and causes necrotizing infection of the surrounding organs due to vascular invasion with thrombosis, subsequently spreading from the sinuses to the orbit and sphenoid sinus and eventually intracranially, causing fatality. Symptoms include more severe headaches than those associated with sinusitis and neurologic symptoms such as rapidly progressing visual impairment, depending on the site of fungal invasion (*7*). Invasive sinonasal fungal disease was once considered a rare disease; however, the number of reported cases has increased. This change could be because of an increased number of patients with conditions that decrease immunity, such as diabetes, long-term steroid administration, and anticancer drug treatment (*2*,*8*). In this case, the underlying disease was poorly controlled; therefore, the infection spread from the sphenoid sinus into the cranium, causing headaches and visual and hearing impairments.

A combination of surgery, systemic antifungal medications, and immunodeficient state improvement is recommended for treating invasive sinusitis (*9*). However, thorough removal is difficult if the infection has spread to the intracranial area, internal carotid artery, or cavernous sinus. Voriconazole is recommended as an antifungal drug for aspergillosis. In contrast, L-AMB is typically used for treating mucormycosis. Although the effective antifungal drug for *B. adusta* remains unclear, in this case, L-AMB treatment was ineffective; therefore, voriconazole was administered, and the patient recovered. After 6 months of treatment, no MRI findings suggested relapse. β-D-glucan levels also decreased, which may reflect the disease course.

The first limitation of this study is that, although *B. adusta* was detected in the nasal biopsy specimen, mixed infections with other causative organisms of invasive sinusitis could not be ruled out. However, no other fungi were detected in the 2 tissue cultures, and we believe that *B. adusta* most likely led to invasive sinusitis. Second, *B. adusta* cannot be tested for drug susceptibility. Although we observed an improvement in the symptoms and laboratory findings after changing the treatment regimen from L-AMB to voriconazole, we cannot confirm that voriconazole is an effective antifungal drug against *B. adusta*.

In summary, we report a case of chronic invasive sinusitis caused by *B. adusta* in an immunocompromised patient with uncontrolled type 2 diabetes. The patient appeared to recover with a treatment regimen consisting of voriconazole. Clinicians should be aware that immunodeficient patients may experience invasive infections because of rare causative fungi.

Appendix Additional information about *Bjerkandera adusta* fungi as causative agent of invasive chronic rhinosinusitis.
