# Supplementary material for: Bjerkandera adusta Fungi as Causative Agent of Invasive Chronic Rhinosinusitis
Source: Emerg Infect Dis. 2025 Feb;31(2):355–8. doi: 10.3201/eid3102.241275 (PMC11845138; doi:10.3201/eid3102.241275)
Supplement: Appendix — Additional information about Bjerkandera adusta fungi as causative agent of invasive chronic rhinosinusitis. [file 24-1275-Techapp-s1.pdf]

Article DOI: https://doi.org/10.3201/eid3102.241275

EID cannot ensure accessibility for supplementary materials supplied by authors. Readers who have difficulty accessing supplementary content should contact the authors for assistance.

Bjerkandera adusta Fungi as Causative Agent of Invasive Chronic Rhinosinusitis

Appendix

Methodology

The colonies of the isolate appeared white and flat when cultured on potato dextrose agar at 25°C for 7 days and did not exhibit any distinctive morphological characteristics at the species level.

DNA extraction, amplification, and sequencing of the internal transcribed spacer (ITS) region were performed following the method of White et al (*1*).

Based on a BLAST search (https://www.ncbi.nlm.nih.gov/search/all/?term = BLAST) of the ITS region sequence, the isolate exhibited a homology of 99.8%–100% with certain strains of *Bjerkandera adusta*. The DNA sequence of the ITS region was submitted to DNA Data Bank of Japan (DDBJ) with the accession number: LC848845. Phylogenetic relationships among the causative isolate, three strains with high homology, and strains related based on ITS region sequences are shown in Appendix Figure 1. The nucleotide sequences were aligned and manually edited using MEGA 11 (Molecular Evolutionary Genetics Analysis version 11). Maximum likelihood (ML) phylogenetic trees were constructed using MEGA 11, based on the general time reversible (GTR) model with gamma distribution (+G) and invariable sites (+I), as recommended by the Model Test. Gaps were completely dilated. Consequently, phylogenetic

analysis revealed the presence of *B. adusta* . It has been preserved as IFM 68197 at the Medical Mycology Research Center, Chiba University, as part of the National Bio-Resource Project in Japan.

References

1. White TJ, Bruns T, Lee S, Taylor J. Amplification and direct sequencing of fungal ribosomal RNA genes for phylogenetics. In: PCR protocols: A guide to methods and applications. Academic Press; 1990. p. 315–322.

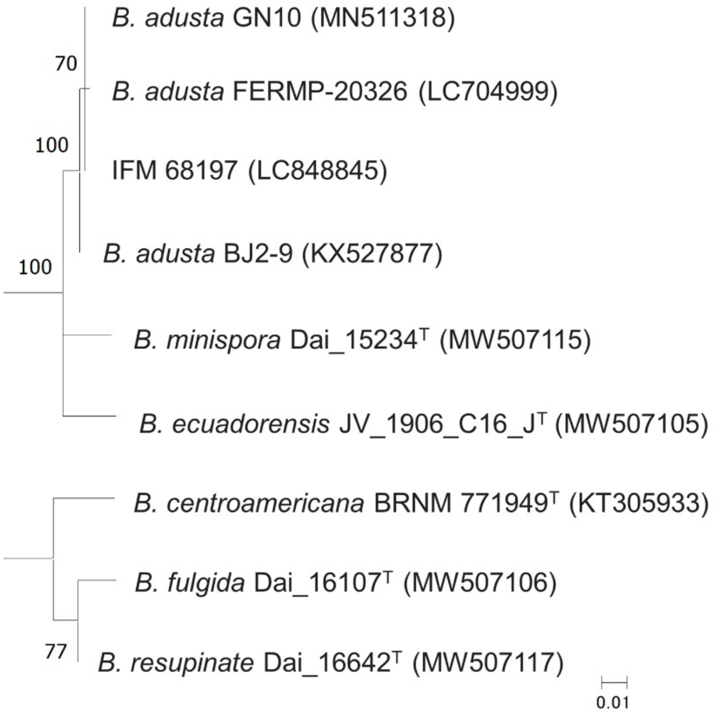

Appendix Figure. Phylogenetic relationships for the causative strain and the related strains based on internal transcribed spacer (ITS) region sequences. A maximum likelihood phylogenetic tree was constructed using MEGA 11 based on the GTR + G + I model, recommended by the Model Test. The numbers on the branches represent bootstrap values exceeding 50% based on 1000 replicates. T: ex-type strain of the species.
